# Merkel Cell Carcinoma: An Immunotherapy Fairy-Tale?

**DOI:** 10.3389/fonc.2021.739006

**Published:** 2021-09-23

**Authors:** Enrica Teresa Tanda, Agostina Lagodin d’Amato, Giovanni Rossi, Elena Croce, Andrea Boutros, Federica Cecchi, Francesco Spagnolo, Paola Queirolo

**Affiliations:** ^1^ Genetics of Rare Cancers, Department of Internal Medicine and Medical Specialties, University of Genoa, Genova, Italy; ^2^ Medical Oncology, Istituto Di Ricovero e Cura a Carattere Scientifico (IRCCS) Ospedale Policlinico San Martino, Genova, Italy; ^3^ Department of Internal Medicine and Medical Specialties (DiMI), School of Medicine, University of Genova, Genova, Italy; ^4^ Medical Oncology, Ospedale Padre Antero Micone, Genova, Italy; ^5^ Department on Medical, Surgical and Experimental Sciences, University of Sassari, Sassari, Italy; ^6^ Division of Medical Oncology for Melanoma, Sarcoma, and Rare Tumors, Istituto Europeo di Oncologia (IEO), European Institute of Oncology IRCCS, Milano, Italy

**Keywords:** merkel cell carcinoma, immunotherapy, merkel cell polyomavirus, advanced disease, anti-PD-1, neoadjuvant

## Abstract

Merkel cell carcinoma (MCC) is a rare, highly aggressive, neuroendocrine cutaneous tumor. The incidence of MCC is growing worldwide, and the disease-related mortality is about three-fold higher than melanoma. Since a few years ago, very little has been known about this disease, and chemotherapy has been the standard of care. Nowadays, new discoveries about the pathophysiology of this neoplasm and the introduction of immunotherapy allowed to completely rewrite the history of these patients. In this review, we provide a summary of the most important changes in the management of Merkel cell carcinoma, with a focus on immunotherapy and a landscape of future treatment strategies.

## Introduction

The history of Merkel cell carcinoma (MCC) therapy is studied with frustration and poor outcomes to treatments until the introduction of immunotherapy, which has radically changed the therapeutic paradigm of this disease.

The incidence of MCC is slowly but steadily growing worldwide. However, MCC is often misdiagnosed and part of this increase in incidence is probably due to the improvement of diagnostic skills, techniques, and the discovery of new biomarkers (1).

Overall, the highest incidence rate has been recorded in Australia, with 1.6 cases/100,000 (2).

In the US, a recently published epidemiological analysis based on the SEER-18 registry (1) counted 6,600 cases of MCCs diagnosed from 2000 to 2013, with an incidence rate rising from 0.5/100,000 in 2000 to 0.7/100,000 in 2013 and an incidence increase of 95.2% (from 334 cases in to 652), exceeding the 56.5% observed in melanoma. Combining these data with US census population data, the global number of new cases of MCC for 2013 is estimated to be 2,488, while the forecasts for 2020 and 2025 are 2,835 and 3,284–3,500 respectively.

In Europe, univocal data are lacking and the incidence of MCC is derived from smaller epidemiological studies. A population-based study published in 2019, including a population based in Northeast France (3), confirmed the increase in new diagnosis, from a rate of 0.05/100.000 in 1985–1989 to 0.22 in 2010–2013. Similarly, a Dutch study (4) showed a rise in the incidence rate for the period 1993–2016, increasing from 0.17 to 0.59. In these studies, the 5-year survival crude rate of MCC ranged between 38% (3) and 41% (2).

The clinical presentation is typically with a non-painful, solid, rapidly growing, and firm nodule, of red color or violaceous. Its surface can be ulcerated or not, covered by crusts, or surrounded by telangiectasias. The diameter at the time of diagnosis usually ranges from 1 to 2 cm (5) but can easily exceed 2 cm due to its rapid evolution. MCC arises frequently on UV-exposed areas (head and neck, limbs, arms), but it is important not to exclude its possible insurgence on non-exposed areas (6). MCC mostly affects Caucasian, older (median age of insurgence is 76 years), immunosuppressed, and hematological populations. All these characteristics and risk factors have been resumed in the acronymous “A.E.I.O.U.” (Asymptomatic, Expanding rapidly, Immune-suppression, Older than 50 years, UV exposed sites), presented for the first time by Heath et al. in 2008 (5).

MCCs grow quickly and metastasize early, with 26%–36% of lesions having lymph node metastasis at the time of diagnosis and 6%–16% having synchronous distant metastasis (6–8). Overall, a large meta-analysis shows that almost 50% and 33% of patients ultimately develop local recurrence or distant metastases, respectively (9). Survival rates of MCC depend on the stage at presentation and range from 50.5% to 13.5% at 5 years of observation (6).

### Origin of MCC

The histogenesis of MCC is still largely debated (10). Firstly described as a “trabecular carcinoma of the skin” by Toker et al. in 1972 (11), MCC took its name from some structural and immunohistochemical (IHC) features that share with Merkel cells (MCs), in particular the expression of ion channel Piezo 2 (12), cytokeratin 20 (CK20), chromogranin A, synaptophysin, and neuropeptides (13–17). However, the cytological and molecular similarity of a tumor cell with a normal cell cannot be considered, to date, a criterion for affirming its certain derivation; indeed, it has been demonstrated that cells undergo several phenotypic changes during oncogenesis, which can strongly modify their final differentiation profile (18). Accordingly, the acquisition of an MC-like phenotype, including neuroendocrine differentiation, during MCC oncogenesis could explain the similarities between MCs and MCCs (19). An example of this process could be the expression of atonal homolog 1 (ATOH1), a transcription factor shared by specific epithelial precursor of MCs (14) and MCC. Since ATOH1 is observed in MCC, its expression could explain the shared phenotype between MCs and MCCs (20). Interestingly, the expression of ATOH1 could be induced by the genetic ablation of Rb1 and the related Rb family protein p130 (21). Nowadays, the initial hypothesis of the MCC origin from MCs has been almost completely abandoned and several factors argue against the direct derivation from MCs. First, in other organs such as lung, strong data suggest that neuroendocrine carcinoma derives more from epithelial progenitors rather than a neuroendocrine cell (22, 23). Second, MCs are mainly post-mitotic cells and thus have low sensitivity to oncogenic stimuli as the expression of small T antigen (sT) that failed to induce cell proliferation or transformation in a transgenic mouse model (24). Third, MCs are most frequently present in the palm and sole in humans, whereas MCC occurs mainly in sun-exposed areas (head and neck, legs). Moreover, no infection of MCs by Merkel cell polyomavirus (MCPyV) has been reported (25). Finally, in an *in vitro* model, MCPyV pseudovirions could barely infect CK20-positive cells obtained from the fetal scalp (0.8%) (26), which argues against an efficient MCPyV infection triggering MCC oncogenesis in an already differentiated MC. Considering these findings, a non-MC could also be candidate for the ancestry of MCC, and an epithelial non-MC (27) as well as a fibroblastic (26) and B-cell (28) origin has been proposed.

### Pathogenesis

**Figure 1:**
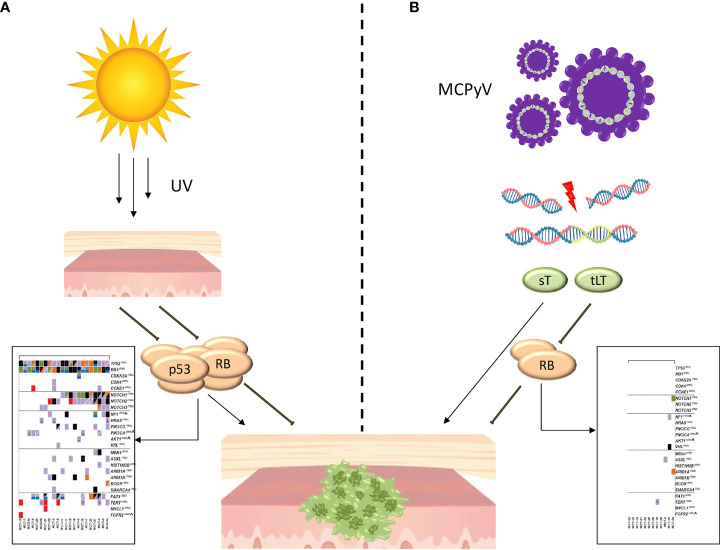
Pathogenesis of MCC. **(A)** Pathogenesis of UV-induced MCC. The progressive DNA damage induced by UV leads to the accumulation of a large number of mutations, largely included in the so-called UV signature, with the most common in p53 and Rb. In the box (29) are reported cancer genes affected by mutation or copy number alterations in UV-induced MCC. **(B)** Pathogenesis of virus-induced MCC. The mutated viral genome is integrated into the host genome, with the expression of two neoantigens: small T (sT) and truncated large T (tLT). The TLT antigen binds to and inactivates Rb while sT antigen inhibits the proteasomal degradation. In the box (29) are reported cancer genes affected by mutation or copy number alterations in virus-induced MCC.

Although many doubts have arisen regarding the cell of origin of MCC, in recent years several discoveries are helping to better define the pathogenesis of MCC, synthesized in **Figure 1**. Currently, the most credited hypothesis is that MCC may be the clinical outcome of two distinct pathogenetic and molecular diseases. In 2008, MCPyV, a member of the polyomavirus family, was discovered to be associated with MCC (30). MCPyV is a small, non-enveloped, double-stranded DNA virus, highly prevalent in the human population (more than 80% among subjects over 50 years old). The virus-related pathogenesis of MCC, illustrated in **Figure 1B**, requires two separate events. First, the circular double-stranded viral genome must be integrated into the host genome, perhaps after a DNA-damaging event. Second, the virus genome must be mutated, with loss of expression of the large T (LT) antigen and the expression of two neoantigens: small T (sT) and truncated large T (tLT). TLT antigen binds to and inactivates Rb, promoting cell-cycle progression and uncontrolled proliferation. ST antigen inhibits the proteasomal degradation. Both tLT and sT demonstrated to drive transformation in mammalian cells *in vitro*; however, numerous attempts to generate mouse models of MCC only partially emulated the disease. These data indicate that additional, as yet undetermined factors are required for induction of MCPyV-associated MCC (31–34). After the integration, host cells start to transcribe and express the MCPyV-related oncoproteins. This is an important phenomenon because the continuous expression of MCPyV oncoproteins is a required factor for survival of virus-positive MCC cells (35), but, at the same time, these persistently expressed non-self antigens elicit host immune recognition with the activation of T-cells and the production of humoral antibodies (36, 37). Interestingly, MCC-specific antibody titers correlate with tumor burden and, consequently, with the response to treatment (38, 39). Eighty percent of MCC in the northern hemisphere is due to the MCPyV viral infection. The remaining 20% seems to be the result of progressive DNA damage induced by UV (**Figure 1A**). Indeed, virus-negative MCC is the solid neoplasm with one of the highest tumor mutational burdens (including melanoma and NSCLC) (40). In most cases, these mutations can be inscribed in the so-called UV-signature mutations (29). The most common are in p53 (75%) and Rb (67%) and commonly result in loss of functional protein expression (41). In conclusion, two distinct pathogenic profiles of MCC have been described. Virus-positive tumor presents a low mutational burden, an antibody titer that correlates with tumor burden, a high PD-L1 expression, and a high TIL level. On the other hand, virus-negative MCC presents a high mutational burden with a median of 1121 mutation/esome, a variable PD-L1 expression and a variable TIL level. All these characteristics form the molecular and biological background that leads to the known sensitivity of this tumor to immunotherapy.

## Treatment of Primary Tumor

MCC being a rare disease, there is a lack of prospective clinical studies, and therefore the studies mostly derive from retrospective analyses.

Surgery is generally considered the first approach, especially in patients with local or regional disease (42–44). Resection margins for primary MCC are not well defined. Guidelines recommend 1- to 2-cm margins with the aim of removing microscopic satellite metastases (43).

Nonetheless, in a retrospective study published in 2018, it was found that a 1-cm margin did not increase the risk of local recurrence in respect to the 1–2-cm margin, and a more radical surgery did not have a significant impact in terms of disease-specific survival or overall survival, but increased the need for a graft or flap closure (45). However, the absence of a statistically significant difference could be explained with the practice to perform wider excision among the most aggressive-appearing lesions.

In another recent retrospective French trial (46), 214 patients were radically resected on the primary site. Among them, 58 (27.1%) had 0.5–1-cm margins and 156 (72.9%) had wider margins (> 1 cm). With a median follow-up of 50.7 months, 5-y OS was 76.8% and 76.2% respectively. Also in this case, there are several limits: the retrospective nature of the trial, the heterogeneous characteristics of the two groups of patients, and the use of radiotherapy as adjuvant treatment after surgery.

On the other hand, in a retrospective trial performed on 79 patients affected by stage I–II MCC, 1-y disease-free survival (DFS) was 51.3%, 71.4%, and 87.8%, while 3-y OS was 57.7%, 82.6%, and 100% among patients with margin < 1 cm, between 1 and 1.9 cm, and ≥2 cm, respectively (47).

Finally, in a recently published retrospective trial (48), 188 stage I–II MCCs were analyzed. A total of 48 patients were treated with surgery alone and, among them, 35 had narrow margins (≤1 cm) while 13 had margin > 1 cm. In the first group of patients, 7 (20%) developed local recurrence, while in the second group, 0 patients developed local recurrence. A group of patients underwent surgery plus RT: this group tended to present higher-aggressiveness tumors or a higher-risk profile (e.g., immunosuppressed) but had less local recurrence than those who were treated with surgery alone (1% vs. 15%), regardless of surgical margins.

As a reasonable conclusion, we can assert that a radical surgery should be performed when possible and that narrow margins could be appropriate if combined with tumor-bed RT.

As we previously mentioned, because MCC is a very radiosensitive cancer, there is the opportunity of a subsequent step with adjuvant radiotherapy on the tumor bed. Indeed, RT demonstrated to improve not only locoregional tumor control but also overall survival in stages I and II, compared with surgery alone (49, 50). In a large, multicenter, retrospective cohort study, 6,156 stage I–II MCC patients who underwent local excision were analyzed (51). In this study, margins > 1 cm were associated with a statistically significant improvement of OS (HR 0.88), with a 5-y OS of 89.8% vs. 76.7% among patients who had local excision with closer margin (≤ 1 cm). In addition to that, radiotherapy induced a statistically significant increase in OS, regardless of surgical margins: patients with close margins who performed RT (HR, 0.81; CI, 0.74–0.89) obtained an OS rate comparable to patients who performed a wider local excision and no RT (HR, 0.80; CI, 0.71–0.89). A systematic review and meta-analysis specifically evaluated the impact of RT in terms of OS and DFS (50). A total of 17,179 cases were analyzed, finding a significant difference in OS (HR 0.8) and in DFS (HR 0.45) between RT and no-RT groups. At the same time, it was found out that local RT does not improve distant metastasis-free survival (DMFS).

RT should be performed as soon as possible after surgery (44), because delay seems to be associated with worse outcome (52). However, results of clinical trials are discordant about the correct timing of RT and in a large retrospective trial that counted 5,952 patients from the National Cancer Database (53); no difference in OS was seen between patients who underwent to RT within 4 weeks and up to 18 weeks.

Sometimes, radical excision may not be feasible, especially in the head/neck region and in elderly patients with poor performance status. In these cases, exclusive radiotherapy should be considered (54–56). In a retrospective trial published in 2021 (55), a total of 84 patients who were treated with either surgery with wide margins (2 cm) plus adjuvant RT (31, 36.9%) or RT alone (53, 63.1%) were analyzed. In these two groups, the local relapse rate was 13.7% in the RT group and 25.8% in the surgery plus RT group, without a statistically significant difference in terms of local or distant relapse and in OS.

### SLNB and Treatment of Regional Lymph Node

In patients without clinically evident nodal disease, NCCN guidelines recommend to perform sentinel lymph node biopsy (SLNB) whenever feasible, no matter the size of the primary tumor (43, 44). The rate of positivity ranges between 11% and 57% and the size of tumor do not seem to correlate with SLN positivity (57–59). The pathological status of lymph nodes is very important to define the prognosis of a patient. A retrospective trial performed on 9,387 patients aimed to validate and refine the AJCC system (8^) showed a 5-y OS of 35.4% among 2,465 patients with nodal metastases (6). Moreover, a difference in terms of OS between patients with clinically negative and clinically positive lymph node metastases was found. Among patients without clinically evident but pathologically proven node metastases, 5-y OS was 39.4%, while for clinically detected lymph node metastases 5-y OS was 26.8%. Moreover, the difference in survival between patients with clinically negative and pathologically negative was 17.8% for T1 tumors (45% *vs*. 62.8%) and similar results were observed among T2, T3, and T4 tumors.

If the presence of micro-metastasis is confirmed, a nodal dissection and/or radiotherapy to the nodal basin is recommended (44). Adjuvant radiotherapy alone or adjuvant radiotherapy combined with a complete lymph node dissection was associated with improved OS in a large retrospective study that included 447 patients (60). The best therapeutic algorithm is still to be defined. Several retrospective studies tried to identify the best strategy. Perez *et al.* (61) in a retrospective single-institution study performed on 71 MCC patients, and Lee *et al.* (62) in a prospective study performed on 163 patients, and found no statistical difference between adjuvant RT, lymph node dissection alone, and radiotherapy with lymph node dissection, concluding that RT or complete lymph node dissection (CLND) could be equivalent. However, in 2020 Cramer et al. (60) published a very significant trial with 447 patients affected by T1–T4, cN0 pN1a, and M0 MCCs who underwent observation, CLND, RT, or CLND + RT. After 3 years of observation, OS was 50%, 52.9%, 67.9%, and 79.5%, respectively. In this trial, adjuvant RT significantly improved OS while CLND did not. Finally, another retrospective trial (63) performed on 72 patients and published in 2021 showed that RT improved OS. As in previously mentioned work, patients underwent observation, RT alone, CLND alone, or RT + CLND. In the same way, RT improved outcomes, especially when combined with CLND. As a conclusion, we can assert that in patients fit for surgery, CLND plus RT should be the treatment of choice, while in patients unfit for combination treatment, the choice should be RT alone. This allows, in selected cases, to obviate the lymph node dissection, and thus its complications, such as lymphedema, neurovascular injury, and surgical-site infections (64). Adjuvant irradiation of the lymphatic drainage area demonstrated to improve locoregional control and the 3-year disease-specific survival rate from 48% to 76% (49).

On the other hand, in case of negative SLNB, the therapeutic algorithm is still debated. In several trials, radiation treatment of the nodal basin was not recommended (65, 66), but guidelines suggest to consider it for high-risk patients.

If SLNB is not performed, elective surgery of at least the first draining lymph node level or radiotherapy is suggested (49).

To sum up and take into consideration the absence of a coded algorithm, the therapeutic approach of each case of MCC should be discussed by a multidisciplinary group consisting of at least an oncologist, a dermatologist, a surgeon, and a radiotherapist (67, 68).

## Systemic Therapy for Advanced Patients

Traditionally, MCC is considered a chemosensitive tumor (69–73). However, chemotherapy (CT) has shown to induce a non-durable response, without a clear benefit in OS and with heavy toxicities (**Table 1**). Due to the rarity of the disease, no specific chemotherapeutic schemes have ever been developed, adopting all therapeutic strategies from small cell lung cancer (SCLC), a tumor that shares several characteristics with MCC.

**Table 1:** Clinical outcomes in selected chemotherapy studies for patients with Merkel cell carcinoma.

	Voog et al. (69)	Tai et al. (70)	Cowey et al. (73)	Iyer et al. (71)
**Setting**	Locally advanced (LA)/metastatic (MTS)	Locally advanced (LA)/metastatic (MTS)	Metastatic (MTS)	Metastatic (MTS)
**Patients (N.)**	69 LA	204	67 I line	62
	72 MTS		20 II line	62 I line
				30 II line
**ORR I L**	61%	59%	31.3%	55%
	69% LA			
	57% MTS			
**mPFS I L**	–	–	4.6 months	3.1 months
**mOS I L**	24 months LA	21.5 months	10.2 months	9,5 months
	9 months MTS			
**5-y OS**	35% LA	17%	24.5% (2-y OS)	–
	17% MTS			
**ORR II L**	45%	–	20%	23%
**mPFS II L**	–	–	2.1 months	2 months
**mOS II L**	–	–	4.4 months	5.7 months
**Toxic death**	7.7% (I line)	3.4%	–	0%

ORR, overall response rate; mPFS, median progression-free survival: 5-y, 5 years; mOS, median overall survival; I L, first line; II L, second line.

Overall, data from a systematic review of literature that analyzed the benefit of CT in advanced MCC showed an ORR ranging from 20% to 61%, higher in the first line than in the second line, and a duration of response (DOR) shorter than 8 months (72). Voog et al. (69) published an analysis of the literature that analyzed data of 107 patients (29 locally advanced and 72 metastatic MCC) treated with several schemes of CT. Here, ORR was 69% among locally advanced and 57% among metastatic MCCs, with a high rate of toxic death in the first line (7.7%). Median OS was 24 months among locally advanced and 9 months among metastatic MCC, with an estimated 5-y OS of 35% and 17%, respectively. ORR in patients receiving second-line chemotherapy was 45%. In another retrospective study (71), 62 metastatic MCC patients were analyzed. All patients were treated with chemotherapeutic schemes, with platinum plus etoposide being the most common choice in the I line. In this analysis, ORR was 55%, with 13% of CR and 42% of PR, and disease control rate (DCR) was 61%. Median progression-free survival (PFS) was 94 days (3 months), and median OS 9.5 months. ORR in the second-line setting was 23% with a median PFS of 61 days (2 months). Finally, in a real-world study published in 2017 (73), data from 67 patients treated with CT in the first line and 20 patients treated in the II line were collected. In the I line group, ORR was 31.3% with a median PFS of 4.6 months and a median OS of 10.5 months. In the second-line group, ORR was 20% (CR = 0%) with a median PFS of 2.1 months and a median OS of 4.4 months.

In conclusion, we can affirm that CT could induce rapid and intense response in MCC patients, but response is not durable, in line with the ability of MCC to quickly develop resistance to CT. Moreover, CT has shown a high rate of toxic death, probably due to the population affected by MCC, often of old age and with severe comorbidities.

The therapeutic scenario in MCC radically changed with the introduction of immunotherapy.

MCC has long been considered a tumor linked, in some way, to the state of activation of the immune system (74). In particular, in support of this hypothesis there was the different incidence of MCC between the immunocompromised and immunocompetent population (3) and case reports of spontaneous regression of MCCs (75), likely due to a T-cell-mediated immune response. Moreover, increasing knowledge of pathogenesis of MCC has highlighted that both virus-induced MCC and UV-induced MCC had the biological rationale to respond to immunotherapy: in the first case, due to the infectious process (**Figure 1**), the production of oncoproteins, and the development of an active immune response; in the second case, due to the presence of a very high mutational burden. On this wave, and with high expectations, trials with immunotherapy in patients affected by MCC have begun to be conducted with the approval of three different agents, two PD-1 inhibitors and one PD-L1 inhibitor. Both these agents act to inhibit the link of the programmed death-ligand 1 (PD-L1) with its receptor, programmed cell death protein 1 (PD-1), normally involved in the suppression of the immune system.

**Figure 2:**
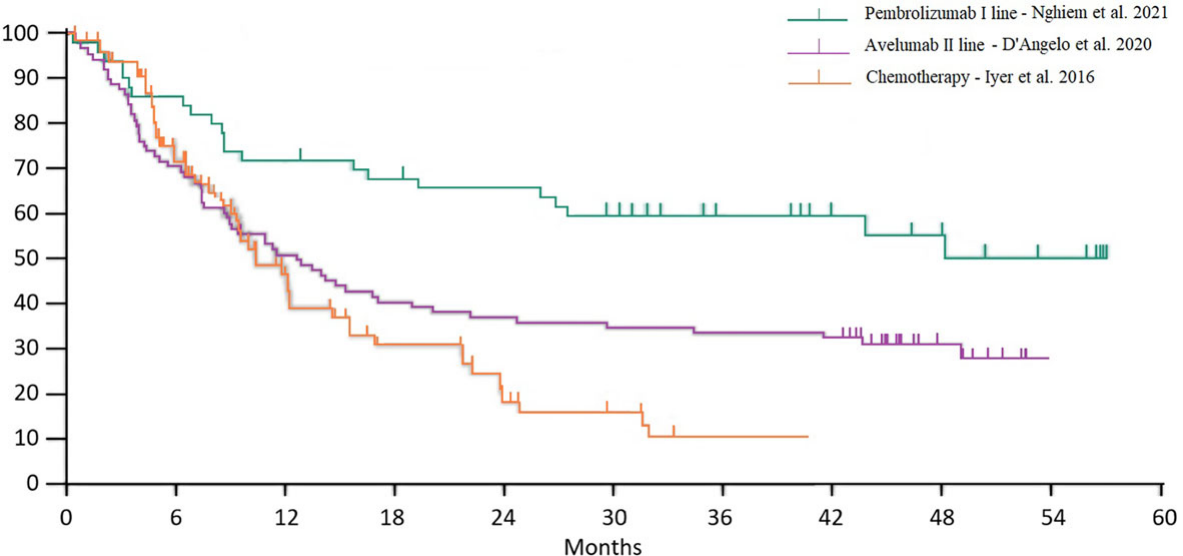
Historical comparison between chemotherapy and immunotherapy overall survival curves.

Food and Drug Administration (FDA) approval of pembrolizumab, nivolumab, and avelumab took place on the basis of three phase II trials. Overall survival curves from studies with chemotherapy and immunotherapy (avelumab second line and pembrolizumab first line) are reported in **Figure 2**. Of note, the populations included in these three trials were substantially different in terms of stage and previous treatments, so the purpose of this extrapolation was to allow a historical and indirect comparison, whereas a direct comparison has never been made in clinical trials.

The Immunotherapy Trials Network (CITN)-09/KEYNOTE-017 study has been a phase II, open-label, non-randomized, multicenter trial involving 50 patients affected by metastatic (m) (86%) or locally advanced (la) (14%) MCC not amenable to definitive surgery or radiotherapy (76). Eligible patients were treated with the anti-PD-1 pembrolizumab at a dosage of 2 mg/kg given intravenously every 3 weeks for up to 2 years or until the development of progressive disease (PD), unacceptable toxicity, or withdrawal of the consent. Patients who showed a progression of the disease were allowed to continue therapy beyond progression if they had a clinical benefit from the treatment. Twelve (24%) patients completed 2 years of treatment. The first analysis performed on 26 patients with a median follow-up of 33 weeks was published in 2016 (76). In this analysis, the ORR was 56%, with 4 CR and 10 PR. Neither PD-L1 expression (on tumor cells or on infiltrating immune cells) nor intratumoral CD8 T-cell infiltration nor viral status of MCC correlated significantly with clinical response to pembrolizumab. The subsequent update (77) considered a total of 50 patients with a median follow-up of 14.9 months. In this report, the ORR was 56%, with 12 CR and 16 PR. Median PFS was 16.8 months, and the estimated 24-mo PFS rate was 48.3%. Median OS had not been reached, while the estimated 24-mo OS was 68.7%. Again, PDL1 expression did not correlate with response and just a trend toward improved OS and PFS in patients with PD-L1 positivity greater than a 1% threshold on tumor cells was observed, but this did not reach statistical significance. On the wave of these results, on December 2018 the FDA granted accelerated approval of pembrolizumab for patients with locally advanced or metastatic MCC. The last update of this trial has been recently published and represents the longest observation of a cohort of patients treated with first-line anti-PD-1, with a median follow-up of 31.8 months (78). The ORR was 58%, with 15 patients achieving CR and 14 patients PR; median DOR was not reached. The majority of responses (90%) developed during the first 12 weeks from the start of treatment and after 3 years of observation 72.7% of responders maintained the response. Median PFS was 16.8 months, and estimated 3-year PFS was 39.1%; median OS was not reached at the time of the analysis, while estimated 3-year OS was 59.4%. When considering only the cohort of responders, 3-year estimated OS reached 89.5%, suggesting that ORR could be considered as an early predictor of OS. In this last update of this trial, factors associated with OS and ORR were analyzed. In detail, the degree of tumor burden reduction, the ability of completing the 2 years of treatment, and an ECOG PS of zero (0) correlated with OS. On the contrary, baseline tumor burden, age, gender, anatomic sites of metastases, tumor viral status, and PD-L status were not associated with ORR or OS. Interestingly, a lower neutrophil–lymphocyte ratio (NLR) during the first 3 months of treatment correlated with outcomes, but the same ratio evaluated at baseline or at any individual time point during the treatment was not statistically significant. Adverse events were substantially consistent with those observed in previous trials with pembrolizumab. Treatment-related adverse events (TRAEs) of any grade were reported in 98% of patients, with 30% of patients reporting grade 3–4 events. Eight patients (16%) discontinued treatment due to TRAEs, and one treatment-related death was reported.

Avelumab is a PD-L1 inhibitor that showed its efficacy in a multicenter, international, prospective, open-label, single-group, phase 2 trial named Javelin Merkel 200 (79). This trial enrolled patients diagnosed with stage IV MCC, refractory to at least a line of chemotherapy. Patient selection was not based on PD-L1 expression or Merkel cell polyomavirus status. Avelumab was given at 10 mg/kg by IV infusion every 2 weeks until disease progression or unacceptable toxicity was confirmed. A total of 88 patients were enrolled. At a median follow-up of 10.4 months, the ORR was 31.8% (28), with 8 CR, 20 PR, and 1 pseudoprogression. Responses were recorded at the first radiological evaluation in 79% of cases, with a median DOR not reached. Median PFS was 2.7 months while median OS was 11.3 months. On the wave of these early results, avelumab was approved by the FDA and EMA. Two subsequent updates were published (80, 81). In the last update, the median follow-up was 40.8 months. At this timepoint, ORR was 33% (29/88 patients), with 10 CR (11.4%). Avelumab seemed to perform better in patients with one previous chemotherapy line in respect to patients treated with two or more lines of chemotherapy (ORR 40.4% vs. 22.2%, respectively), while the sites of metastasis (visceral vs. non-visceral) did not appear to impact on ORR. Among patients whose tumors were assessable for PD-L1 expression (73), ORR was 36.8% in PD-L1-positive (57) and 18.8% in PD-L1 negative (16) patients. Regarding viral status, among 46 virus-positive and 31 virus-negative patients, the ORR was 28.3% and 35.5%, respectively. Such results were in line with a *post hoc* analysis published in the first report of the trial. At the time of the last analysis, responses were ongoing in 17 of 29 responders (58.6%) regardless of PD-L1 status, with 4 patients who maintained the response for more than 3 years. Median DOR was 40.5 months. PFS at 2 and 3 years of observation was 26% and 21%, respectively, while median OS was 12.6 months, with a 3- and 4-y OS of 32% and 31%, respectively. TRAEs of any grade occurred in 62 (70%) patients, with a particularly high rate of infusion reaction (17%) that induced to recommend the use of a premedication with H1-antihistamine and paracetamol 30–60 min before avelumab treatment; grade 3 TRAEs were reported in four (5%) of 88 patients. Two patients (2%) permanently discontinued treatment because of an adverse event. In this paper, exploratory biomarker analysis data were reported. Several factors were evaluated, but no single biomarker was consistently associated with a clinical benefit. Best outcomes were recorded among high TMB, virus-negative, or PD-L1-positive (or with a high level of TILs) patients that received just one prior systemic therapy.

Avelumab as a first-line treatment was evaluated in part B of Javelin Merkel 200 (82). Here, 39 stage IV chemo-naïve MCC patients were treated with avelumab upfront. Data from an interim analysis of this trial were reported in 2018, with a median follow-up of 5.1 months. At the time of the analysis, treatment was ongoing in 24 patients (61.5%), while 15 (38.5%) discontinued due to PD (7%–17.9%), adverse events (6%–15.4%), or death (2%–5.1%). Efficacy was evaluated in 29 patients with at least 3 months of follow-up, and in a subgroup of 14 patients with at least 6 months of follow-up. In the 3-month follow-up group, the ORR was 62.1%, with 4 (13.8%) CR and 14 (48.3%) PR, and a DCR of 72.4%. As observed in Javelin Merkel 200 part A and in KN017, 88.9% of responses were observed at the first radiological evaluation. Among responders, 14 (77.8%) patients had an ongoing response at the time of the analysis, with a median DOR not estimable. Median PFS was 9.1 months and the 3-month PFS was 67%. In the 6-month follow-up group, the ORR was 71.4% with 4 (28.6%) CR, 6 (42.9%) PR, and a DCR of 78.5%. Updated data with a median of 21.2 months of follow-up were presented in 2019 during the SITC congress (83). A total of 116 patients had been treated with avelumab, and, at the time of the analysis, treatment was ongoing in 26 patients (22.4%). The ORR was 39.7%, including 19 CR (16.4%) and 27 PR (23.3%), with slightly better results in the PD-L1-positive cohort in respect to the PD-L1 negative cohort (61.9% and 33.3%, respectively), and a median DOR of 18.2 months. Median PFS was 4.1 months with 6- and 12-month PFS rates of 41% and 31%, respectively. Median OS was 20.3 months, and the 12-month OS rate was 60%. In the PD-L1-positive and PD-L1-negative subgroups, 1-y OS rates were 71% and 56%, respectively. The SPEAR-Merkel study has been published in 2021 and reported clinical outcomes in patients affected by locally advanced or metastatic MCC treated with avelumab first line, in a real-world setting (84). A total of 36 patients were enrolled, 28 (32.1%) with laMCC and 19 (67.9%) with mMCC. Two-thirds of the overall 1L avelumab population (64.3%) discontinued 1L avelumab during the study period due to disease progression (33.3%), physician preference (27.8%), toxicity, or not documented (11.1% each). ORR was 64.3% (66.7% in laMCC and 63.2% in mMCC) with nine complete responses (three laMCC and six mMCC). The median DOR was 15.5 months, NR in patients with laMCC, and 9.6 months in patients with mMCC. The median PFS was 11.4 months, and the median OS was 20.2 months. Neither the median PFS nor the median OS was reached in patients with laMCC. In patients with mMCC, the median PFS was 10.0 months, and the median OS was 20.2 months. All results were consistent with data from the registration trial.

Data from the subsequent Expanded Access Program (EAP) program were published in August 2020 (85). In the EAP, patients who progressed after at least one line of chemotherapy and chemo-naïve patients who were ineligible for chemotherapy (evaluated case by case) were enrolled. Patients were not selected based on tumor PD-L1 expression or MCPyV status. A total of 494 patients were treated, including 15 who received treatment as a first line. Response data were available for 254 patients, and outcomes were provided for 240 patients. Results were substantially consistent with those from registration trials, with an ORR of 46.7%, including CR in 22.9%, PR in 23.8%, and a DCR of 71.2%. The safety profile was further confirmed, and avelumab showed a toxicity spectrum very similar to other anti-PD-1/PD-L1, except for infusion-related reactions, which occurred in nine patients. The relatively high number of infusion-related reaction deserves the recommendation to use a premedication with paracetamol and antihistaminic for at least the first four cycles of avelumab.

Finally, in July 2017 the results of the anti-PD-1 nivolumab were published (86). Nivolumab was evaluated among patients with five types of advanced virus-associated cancers who had received ≤2 prior therapies. At a median follow-up of 26 weeks, among 25 MCC patients who received treatment, 22 were evaluable for response, with an ORR of 68% and ongoing responses in 13 of 15 (87%) patients. Responses occurred in treatment-naive patients (71%), in patients with one to two prior systemic therapies (63%), and in both virus-positive and virus-negative tumors; 67% of responses occurred at ~8 weeks. At 3 months, PFS and OS rates were 82% and 92%, respectively.

The characteristics and results of all trials with immunotherapy for the treatment of advanced MCC are summarized in **Table 2**.

**Table 2:** Summary of all clinical trials with immunotherapy for the treatment of locally advanced and/or metastatic MCC.

	KN 017 (78)	Javelin Merkel 200 (part B) (83)	Javelin Merkel 200 (part A) (81)	CM – 358 (86)
**Drug**	Pembrolizumab	Avelumab	Avelumab	Nivolumab
**Line**	I	I	≥II	≥II
				I
**MCC status**	Locally advanced/metastatic	Metastatic	Metastatic	–
				–
**N. pts**	50	116	88	8
				14
**F.U. (mo)**	31.8	21.2 mo	40.8 mo	26 weeks
**ORR % (n)**	58 (29)	39.7 (46)	33 (29)	63%
				71%
**CR % (n)**	30 (15)	16.4 (19)	11.4 (10)	0 (0)
				21 (3)
**DCR % (n)**	66 (33)	–	43.2 (38)	76 (6)
				71 (10)
**PFS**	m: 16.8 mo	1-y: 31%	m: 3 mo	3-mo: 82%
	3-y: 39.1%		3-y: 21%	
**OS**	m: NR	m: 20.3 mo	m: 12.6 mo	3-mo: 92%
	3-y: 59.4%	1-y: 60%	4-y 31%	

N.pts, number of patients; F.U., follow-up; ORR: overall response rate; CR, complete response; DCR, disease control rate; PFS, progression-free survival; OS, overall survival.

## Future Directions for Advanced Disease

Future directions in MCC include several therapeutic strategies, such as immunotherapy, targeted therapies, and epigenetic drugs, in both neoadjuvant, adjuvant, first-line, and subsequent line settings. Indeed, 50% of patients do not adequately respond to anti-PD-L1/anti-PD-1 monotherapy (treatment resistant, or relapsed) and second-line therapy in MCC is still uncoded. To answer this medical need and to give a therapeutic alternative to patients unfit for chemotherapy and absolute contraindication to immunotherapy, several trials with target therapy have been performed and others are currently ongoing. However, most trials with targeted therapies alone had disappointing results. A summary of all trials currently ongoing for advanced MCC is reported in **Table 3**.

**Table 3:** Summary of all available trials for the treatment of locally advanced or metastatic MCC.

NCT	Phase	MCC stage	Drugs	N	Recruitment status	Study outcomes/primary objectives**	Ref.
**NCT02514824**	I/II	IV or recurrent	MLN0128	9	Completed	Negative.	(87)
						Lack of efficacy.	
**NCT02036476**	II	IV or recurrent	Cabozantinib	8	Active, not recruiting	Negative.	(88)
						mPFS: 2,1 mo	
						mOS: 11.2 mo	
						Poor tolerability and lack of activity	
**NCT00079131**	II	III–IV	Oblimersen	37	Completed	Negative	(89)
						ORR = 0%. SD = 3 patients.	
**NCT00068783**	II	III–IV	Imatinib mesylate	40 (23)	Completed	CR = 0; PR = 1; ORR = 4%; SD = 3. mPFS = 1 mo; Estimated 6-mo PFS = 4%.	(90)
						mOS = 5 mo; Estimated 1-y OS = 17%.	
**NCT02351128**	II	III–IV	Lanreotide	35	Completed	DCR 20% (7/35)	(91)
**NCT01652547**	I	IV	Pasireotide	10	Completed	Terminated early due to slow recruitment after 2 y from study initiation. No data on MCC cohort.	(92)
**NCT03787602**	II	III–IV	KRT-232 (MDM2 Antagonist)	46	Recruiting	ORR	
**NCT04276597**	II	III–IV	177Lu-DOTATOC	50	Recruiting	ORR	
**NCT04261855**	I/II	IV	Avelumab, radiation (EBRT), radiation (Lutetium-177 (177Lu)-DOTATATE)	65	Recruiting	PFS at 12 mo	
**NCT02054884**	II	IV	F16IL2, paclitaxel	13	Terminated (lack of enrollment)	ORR	
**NCT04874831**	II	IV	Avelumab, domatinostat	90	Not yet recruiting	ORR	
**NCT04393753**	II	III–IV	Avelumab, domatinostat	40	Recruiting	ORR	
**NCT02035657**	Proof of concept	III–IV	GLA-SE	10	Completed	Safety and feasibility	
**NCT03783078**	III	III–IV	Pembrolizumab	50	Active, not recruiting	ORR	
**NCT04792073**	II	III–IV	Avelumab, radiation	36	Recruiting	PFS at 12 mo	
**NCT03599713**	II	IV or recurrent	INCMGA00012	100	Recruiting	ORR	
**NCT03988647**	II	IV	Pembrolizumab, radiation	1	Active, not recruiting	ORR	
**NCT03167164**	I/II	IV	Avelumab, bevacizumab, capecitabine, Cisplatin, cyclophosphamide, 5-fluorouracil, leucovorin, nab-paclitaxel, omega-3-acid ethyl esters	0	Withdrawn (trial not initiated)	Safety and ORR	
			Radiation (stereotactic, body radiation therapy),				
			ALT-803, ETBX-051, ETBX-061, GI-6301, haNK				
**NCT03853317**	II	IV	Avelumab, N-803, haNK		Recruiting	ORR	
**NCT02465957**	II	III–IV	aNK (NK-92)	24	Active, not recruiting	PFS	
**NCT03228667**	II	III–IV	N-803, pembrolizumab, nivolumab, atezolizumab, avelumab, durvalumab, pembrolizumab, PD-L1 t-haNK	636	Recruiting	ORR	
**NCT01913691**	II	IV	Ipilimumab	0	Withdrawn	OS at 12 mo	
**NCT01758458**	I/II	IV or recurrent	Aldesleukin, MCPyV TAg-specific polyclonal autologous CD8-positive T cells, radiation, recombinant interferon beta	4	Terminated	Safety and median time to new metastasis	
					(A phase I/II study (NCT01758458) is now recruiting)		
**NCT01440816**	II	NA	Tavokinogene telseplasmid (tavo)	15	Completed	Iincresing in expression of IL-12	
**NCT03071406**	II	IV	Ipilimumab, nivolumab, radiation	50	Recruiting	ORR	
**NCT04590781**	I/II	III–IV	Pembrolizumab, XmAb18087	142	Not yet recruiting	Safety and ORR	
**NCT01013779**	II	II–III	Carboplatin, etoposide, radiotherapy	43	Active, not recruiting	Time to locoregional failure and safety	
**NCT02584829**	I/II		Avelumab, recombinant INF beta, radiation, MCPyV TAg-specific polyclonal autologous CD8-positive T cells	8	Active, not recruiting	ORR and safety	
**NCT02819843**	II	III–IV	TALIMOGENE LAHERPAREPVEC (TVEC), radiation (hypofractionated radiotherapy)	19	Active, not recruiting	ORR	
**NCT00003549**	II	III–IV	CMF regimen, cyclophosphamide, fluorouracil, methotrexate	80	Completed	Not avalable	
**NCT04160065**	I	III–IV	IFx-Hu2.0	20	Recruiting	Safety	
**NCT04853602**	expanded access	III–IV	IFx-Hu2.0	-	Recruiting	Not available	
**NCT03684785**	I/II	III–IV	Cavrotolimod, pembrolizumab, cemiplimab	130	Recruiting	Safety	
**NCT03304639**	II	III–IV	Pembrolizumab, radiation (stereotactic body radiation therapy)	100	Active, not recruiting	PFS	
**NCT00346385**	I	IV	BB-10901	97	Completed	Safety	
**NCT03901573**	I/II	IV	NT-I7, atezolizumab		Recruiting	Ssafety	
**NCT02978625**	II	IV	Nivolumab, talimogene laherparepvec	68	Recruiting	ORR	
**NCT03458117**	I	III–IV	Talimogene laherparepvec (T-VEC)	20	Recruiting	Activation of biomarkers	
**NCT00004922**	II	IV	Irinotecan hydrochloride	31	completed	Not available	
**NCT00003514**	II	IV	Antineoplaston A10, antineoplaston AS2-1	0	Withdrawn	Not available	
**NCT03747484**	I/II	III–IV	Autologous MCPyV-specific HLA-A02-restricted TCR-transduced CD4+ and CD8+ T-cells FH-MCVA2TCR, avelumab, pembrolizumab, radiation	16	Recruiting	Safety and ORR	
**NCT03816332**	I	III–IV	Ipilimumab, nivolumab, prednisone, tacrolimus	16	Suspended (scheduled interim monitoring)	Safety	
**NCT02831179**	I	III–IV	Capecitabine, temozolomide, veliparib	0	Withdrawn (loss of funding support)	Maximum tolerated dose	
**NCT03107663**	I	III–IV	^89^Zr-Df-IAB22M2C	15	Completed	Safety	
**NCT01204476**	I	III–IV	Cixutumumab, everolimus, octreotide acetate	27	Completed	mPFS: 43,6 weeks, mOS: 25,5 mo. No data on MCC cohort.	(93)
**NCT03074513**	II	III–IV	Atezolizumab, bevacizumab	164	Active, not recruiting	ORR	
**NCT04234113**	I	III–IV	SO-C101, pembrolizumab	96	Recruiting	DLT	
**NCT03435640**	I/II	III–IV	NKTR-262, bempegaldesleukin, nivolumab	64	Active, not recruiting	Safety	
**NCT03629756**	I	III–IV	Etrumadenant, zimberelimab	44	Active, not recruiting	Safety	
**NCT04725331**	I/II	III–IV	BT-001, pembrolizumab	48	Recruiting	Safety/ORR	
**NCT02890368**	I	IV or recurrent	TTI-621, PD-1/PD-L1 Inhibitor, pegylated interferon-α2a, T-Vec, radiation	56	Terminated	Safety	
**NCT04246671**	I/II	III–IV	TAEK-VAC-HerBy	45	Recruiting	DLT	
**NCT03935893**	II	III–IV	Tumor-infiltrating lymphocytes (TIL), fludarabine, cyclophosphamide	10	Recruiting	DLT	
**NCT04272034**	I	III–IV	INCB099318	100	Not yet recruiting	Safety	
**NCT04242199**	I	III–IV	INCB099280	140	Recruiting	Safety	
**NCT04260802**	I/II	III–IV	OC-001, anti-PD-1/anti-PD-L1	80	Recruiting	DLT	
**NCT03841110**	I	III–IV	FT500, nivolumab, pembrolizumab, atezolizumab, cyclophosphamide, fludarabine, IL-2	76	Recruiting	DLT	
**NCT03652077**	I	III–IV	INCAGN02390	40	Active, not recruiting	Safety	
**NCT03538028**	I	III–IV	INCAGN02385	22	Completed	Safety	
**NCT02643303**	I/II	III–IV	Durvalumab, tremelimumab, poly ICLC	102	Recruiting	PFS at 24 weeks	
**NCT04187872**	I	III–IV	LITT + pembrolizumab	16	Recruiting	Immune effect on blood	
**NCT03212404**	I	III–IV	CK-301 (cosibelimab)	500	Recruiting	DLT	
**NCT01155258**	I	III–IV	Temsirolimus, vinorelbine ditartrate	19	Completed	MDT	
**NCT02479698**	II	III–IV	Allogeneic BK-specific Cytotoxic T-lymphocytes	100	Recruiting	ORR	
**NCT03589339**	I	III–IV	NBTXR3	60	Recruiting	ORR	
**NCT00002947**	I	III–IV	Indium In 111 pentetreotide	35	Terminated	Not available	
**NCT00655655**	I	III–IV	Everolimus, vatalanib	96	Completed	MTD	

When published or presented, outcomes are reported with corresponding reference.

N, number of patients enrolled; Ref, reference; m, median; PFS, progression-free survival; OS, overall survival; ORR, overall response rate; CR, complete response; PR, partial response; SD, stable disease; DCR, disease control rate; DLT, dose-limiting toxicity; MTD, maximum dose tolerated.

**If no results are available, we indicate the primary objectives of the study.

MLN0128 is a second-generation TORC1/2 inhibitor that showed preclinical activity in MCC cell lines, decelerating tumor cell growth, diminishing cell proliferation, inducing apoptosis, and enhancing antitumor effect when combined with JQ1 (a bromodomain protein BRD4 inhibitor) (94). On this wave, a clinical trial with MLN0128 was performed (NCT02514824). The study never passed from phase I to phase II, and no efficacy data are available. From the few data reported, the study was closed due to a lack of efficacy and a slow recruitment (87).

Cabozantinib is a multiple-kinase inhibitor, including c-MET and VEGFR-2, commonly used in the treatment of several metastatic solid cancer. Cabozantinib (88) was evaluated in a prospective phase II trial (NCT02036476) that enrolled eight metastatic or locally advanced platinum-resistant MCC patients. The trial was closed prematurely due to poor tolerability and lack of activity of the study drug, which obtained a median PFS of 2.1 months and a median OS of 11.2 months. Notably, patients were not selected based on the presence of any mutation.

Oblimersen binds to human bcl-2 mRNA-stimulating apoptosis and is believed to facilitate non-apoptotic cell death by autophagy, to inhibit tumor angiogenesis, and to exert immunostimulatory effects. Preclinical studies (95) performed on MC-MA 11 MCC xenografts obtained encouraging results and provided the basis to a Simon two-stage phase II trial to evaluate oblimersen efficacy among MCC patients (89). A total of 12 patients were treated, but ORR was 0% and only 3 patients achieved a SD.

Imatinib was also evaluated as a potential treatment strategy in MCC. On the wave of the identification of c-Kit expression in this neoplasm, a clinical trial with imatinib mesylate was initiated (NCT00068783). Among 23 treated patients, ORR was 4% with 0 CR and 1 RP, and SD was achieved in 3 patients. Median PFS was 1 month with an estimated 6-mo PFS of 4%; estimated median OS and 1-y OS were 5 months and 17%, respectively (90).

Somatostatin analogues (SSAs) are commonly used in low- and medium-grade neuroendocrine tumors (NET), but several studies support their possible use in MCC therapy (96–98). Lanreotide has been evaluated in a phase II study (NCT02351128) on 35 patients (91). Among them, seven (20%) obtained a disease control form more than 3 months. Pasireotide had also been evaluated among melanoma and MCC patients in a phase I trial (NCT01652547). However, no data are available for the MCC cohort (92). In a recently published retrospective trial (96), 40 patients were evaluated for somatostatin receptor (SRS) expression. A total of 33 patients (85%) had some degree of SRS uptake, and 19 patients were treated with SSAs. Among them, seven had a response-evaluable target lesion and three (43%) experienced disease control, with a median PFS of 237 days. The major limit of this study is the confounding effect induced by radiotherapy, which made several lesions not radiologically evaluable according to RECIST. Interestingly, the degree of SRS expression did not correlate significantly with the efficacy endpoints.

Peptide receptor radionuclide therapy (PRRT) with (177) Lu-DOTATATE could be a potentially active therapy in MCC. Several case reports described objective responses in metastatic MCC patients (99, 100), and a phase II trial is currently ongoing (NCT04276597).

Combining targeted therapy and immunotherapy is known to be an interesting and promising strategy in several solid tumors (101, 102). In MCC, a number of clinical trials are ongoing to assess such combination strategy. One of the most promising agents to use in combination is domatinostat, an enzyme histone deacetylase inhibitor (HDAC) able to modulate the tumor microenvironment and to enhance antitumoral immunological response. In a phase I study performed on 24 pretreated patients, affected by several solid cancers, this oral molecule showed a favorable toxicity profile at 200 mg/BID, being able to induce 1 CR, 1 PR, and 18 SD (103). Combination between domatinostat and immunotherapy (pembrolizumab) has been subsequently evaluated in a phase II trial (104) that assessed the safety of this combination and the potentially ability of domatinostat to increase the antitumor activity of pembrolizumab. Currently, two phase II trials with avelumab plus domatinostat are recruiting patients (NCT04874831; NCT04393753).

## Adjuvant and Neoadjuvant Approach

Adjuvant and neoadjuvant approaches are not a current clinical practice. However, several clinical trials are investigating treatments this setting, with interesting results (**Table 4**). The first ADMEC trial (NCT02196961) with adjuvant ipilimumab *versus* observation in resected MCC patients was closed after 22.3 months of follow-up due to a futility analysis showing lack of efficacy and a strong toxicity of ipilimumab (105). Data of the phase II ADMEC-O trial with adjuvant nivolumab (NCT02196961), the phase III ADAM trial (NCT03271372) with adjuvant avelumab, and the phase III STAMP study (NCT03712605) are still awaited. Notably, several clinical trials include very early stage MCC, like stages I and II (see **Table 4**).

**Table 4:** Summary of all available trials currently ongoing for the treatment of completely resected MCC with an adjuvant intent, or potentially resectable MCC with a noeadjuvant intent.

Trial	NCT	Phase	Stage MCC	Drugs	N	Recruitment status	Study outcomes	Ref.
**Ipilimumab adjuvant ADMEC (DeCOG) Ph II, open, randomized vs. observation**	NCT02196961	II	II–III–IV completely resected	Ipilimumab	40	Terminated	Negative. no difference in PFS	(105)
**Adjuvant Therapy of Completely Resected Merkel Cell Carcinoma With Immune Checkpoint Blocking Antibodies vs. Observation (ADMEC-O)**	NCT02196961	II	II–III–IV completely resected	Nivolumab	180	Active, not recruiting	No data	
**Nivolumab and Radiation Therapy or Ipilimumab as Adjuvant Therapy in Treating Patients With Merkel Cell Cancer**	NCT03798639	I	III completely resected	Nivoluamb, Radiation, Ipilimumab	7	Active, not recruiting	No data	
**Adjuvant Avelumab in Merkel Cell Cancer (ADAM)**	NCT03271372	III	III completely resected	Avelumab	100	Recruiting	No data	
**Immunotherapy Adjuvant Trial in Patients With Stage I–III Merkel Cell Carcinoma (I-MAT)**	NCT04291885	II	I, II, III completely resected	Avelumab	132	Recruiting	No data	
**Pembrolizumab Compared to Standard of Care Observation in Treating Patients With Completely Resected Stage I-III Merkel Cell Cancer, STAMP Study**	NCT03712605	III	I, II, III completely resected	Pembrolizumab, radiation	500	Recruiting	No data	
**Neoadjuvant Nivolumab for Patients With Resectable Merkel Cell Carcinoma in the CheckMate 358 Trial**	NCT02488759	I/II	IIA–IV resectable	Nivolumab	39	Active, not recruiting	24 mo-RFS pCR/MPR: 88.9%;	(106)
							24 mo-RFS rPR/rCR: 90.9%;	
							24 mo-OS pCR/MPR: 100.0% and 88.9%	
							24 mo-OS rPR/rCR: 100%	
**Neoadjuvant Lenvatinib Plus Pembrolizumab in Merkel Cell Carcinoma**	NCT04869137	II	II–III–IV resectable	Pembrolizumab, lenvatinib	26	Recruiting	No data	

N, number of patients; ref, reference; RFS, Relapse Free Survival, pCR/MPR, pathologic complete response/major pathologic response; OS, Overall Survival.

A neoadjuvant approach was explored in CheckMate 358 (106), a phase I/II study that enrolled 39 patients affected by completely resectable MCC (stages IIA–IV). A total of 36 patients received 2 cycles of neoadjuvant nivolumab, followed by surgery. Pathological response (pR) and radiological response (rR) were correlated with clinical outcomes. All patients were evaluated for pR by study investigators, while a total of 26 patients were evaluated by central pathologic review, finding a pathological complete response rate (pCR) of 47.2% (n = 17) and 46.2% (n = 12), respectively; among patients evaluated centrally, the major pathological response (MPR) rate was 15.4% (n = 4). A total of 33 patients were radiologically evaluable, with an ORR of 54.4% (n = 18). Notably, radiographic response seemed to underestimate the degree of pR: indeed, among 11 rR < 30% (non-CR, non-PR), 5 had pCR; moreover, rCR has been significantly less than pCR. Median recurrence-free survival (RFS) and median OS were not reached at 20.3 months of follow up, while 24-month RFS and 24-month OS were 68.5% and 79.4% in the whole population, respectively. Both pR and rR correlated with RFS and OS. Indeed, 24-month RFS among patients that had a pCR/MPR by central review and among patients who obtained at least an rPR were 88.9% and 90.9%. In the same way, 24-month OS among patients who developed a pCR by central review, or at least an rPR, was 100.0%. A neoadjuvant study with pembrolizumab plus lenvatinib (NCT04869137) is currently recruiting patients.

## Discussion

Treatment of MCC is an emerging issue in everyday clinical practice. If in the past years this tumor was considered as a sort of SCLC in terms of biological behavior and clinical management, today it has become an object of numerous studies. Indeed, until recently, standard treatment was based on chemotherapeutic schemes with disappointing results, with a median survival of 9–10 months (69–73). Currently, the standard of care for the treatment of this neoplasm is immunotherapy with avelumab (anti-PD-L1) which received FDA and EMA approval, and pembrolizumab and nivolumab which was approved for the same indication by the FDA only. First-line pembrolizumab in locally advanced and metastatic MCC achieved a median OS not reached at a median follow-up of 31.8 months, and a 3-y OS of 59.4% (78), while first-line avelumab in metastatic MCC showed a median OS of 20.2 months (83). In pretreated patients progressing to chemotherapy, avelumab showed a median OS of 12.6 month and a 4-y OS of 31% (81).

The fact that immunotherapy performs worse in the second-line setting rather than in the first line is likely to depend on the type of patient, classically fragile, elderly, and with severe comorbidities, whose conditions tend to a progressive worsening, and on the biology of this disease which is characteristically very aggressive. Therefore, in patients with no absolute contraindications to immunotherapy, upfront treatment with anti-PD-1/anti-PD-L1 agents is recommended. A high burden of disease and/or the presence of clinical symptoms do not contraindicate the initiation of upfront immunotherapy. Indeed, it has been shown that immunotherapy is able to induce rapid responses, most of them observed at the first radiological evaluation, lasting over time (78, 83). Starting the therapeutic strategy with a chemotherapy treatment has shown, in a retrospective study, to cause a substantial reduction of patients who will be able to receive second-line treatment, a reduction of the duration of the first line itself, and a reduction of the time to second-line initiation, due to the rapid progression observed in the course of chemotherapy (107).

Until today, no predictive factors for anti-PD-L1/PD-1 therapy are accepted, although tumor PD-L1 expression, virus status, and some other factors may correlate. Tumor PD-L1 expression (PD-L1 negative versus PD-L1 positive) seems to correlate with efficacy of immunotherapy, in line with results observed in other tumor types. However, no definite conclusions have been drawn.

The second line in MCC remains an unmet medical need.

Indeed, almost 50% of patients do not respond to anti-PD-L1/anti PD-1 and, at the time of the disease progression, few therapies are easily available other than chemotherapy. The motivation for this choice is twofold. First, chemotherapy has a high ORR and often these patients progress rapidly and with high disease burdens: chemotherapy allows us to reduce tumor burden, partially improving the patients’ quality of life. Second, due to the rapid kinetic of this tumor, the survival of these patients in the absence of treatment (best supportive care) is extremely low and chemotherapy, although with known limits, allows us to obtain some advantages. Clinical practice involves the use of standard chemotherapy schemes such as platinum in combination with etoposide.

There are currently no recruiting trials for patients progressing from anti-PD-1/anti-PD-L1 therapy, and this is certainly a major limitation to the therapeutic prospects for patients under treatment. In our opinion, it would be appropriate to start second-line trials, for example to evaluate the effectiveness of the continuation of anti-PD-1 in association with standard chemotherapy. This approach has already given positive results in SCLC, a neoplasm that shares several characteristics with MCC in terms of clinical and biological behavior, tumor kinetic, and sensitivity to chemotherapy. Indeed, carbo/cis-platinum plus etoposide plus anti-PD-L1 as a first line of treatment has been evaluated in Caspian and Empower 133 trial (108, 109) and showed a good safety profile and improved efficacy in terms of OS and PFS in respect to chemotherapy alone. To date, a similar approach in MCC remains completely unexplored in the first and second lines.

Numerous trials are evaluating strategies with molecularly targeted drugs. After some disappointing results with cabozantinib (88) and oblimersen (89), new hopes are now placed in treatment with somatostatin analogues. Indeed, encouraging data from case reports and case series are currently available, as well as from a small phase II study with lanreotide, which showed a DCR of 20% (91, 92, 98). Larger and more standardized clinical trials will be needed to define the real benefit of these treatments.

As we reported before, immunotherapy provides a clinical benefit in approximately 50% of patients, with the aim to increase the percentage of responders, overcome the mechanisms of primary resistance, and prevent the development of secondary resistance, like MHC-I downregulation, low CD8 T cell response, and Th2 polarization of CD4 T cells (110, 111). One of the most promising agents is domatinostat, which showed a favorable toxicity profile in a phase I trial and promising results in combination with pembrolizumab in a phase II trials (104). Currently, two phase II trials with avelumab plus domatinostat are recruiting patients (NCT04874831; NCT04393753). The adjuvant/neoadjuvant approach is currently not part of everyday clinical practice, but it is an extremely promising field of research. The very positive results of the CM 358 study with nivolumab in the neoadjuvant setting (106) showed the great potential of this therapeutic strategy and numerous trials are being developed to define the role of a possible early treatment in MCC. In CM 358, the pathological complete response rate and the major pathological response rate were 46.2% and 15.4%, respectively. Notably, pathological complete response rates in neoadjuvant anti-PD-1 trials in NSCLC and in melanoma were 15% and 19%–25% (112, 113). In light of these preliminary results, there is high expectation for the currently ongoing trials with adjuvant nivolumab, adjuvant avelumab, and neoadjuvant pembrolizumab plus lenvatinib.

## Author Contributions

ET conceived the review focus, conducted the literature review, summarized the manuscript, analyzed the data, wrote the first draft, and finalized the manuscript. FS and PQ coordinated and supervised the review. ALd’A, GR, EC, AB, FC, FS, and PQ reviewed the literature and revised and made corrections to the manuscript. All authors contributed to the article and approved the submitted version.

## Conflict of Interest

The authors declare that the research was conducted in the absence of any commercial or financial relationships that could be construed as a potential conflict of interest.

## Publisher’s Note

All claims expressed in this article are solely those of the authors and do not necessarily represent those of their affiliated organizations, or those of the publisher, the editors and the reviewers. Any product that may be evaluated in this article, or claim that may be made by its manufacturer, is not guaranteed or endorsed by the publisher.
